# Presenting wicked problems in a science museum: A methodology to study interest from a dynamic perspective

**DOI:** 10.3389/fpsyg.2023.1113019

**Published:** 2023-02-10

**Authors:** Rooske K. Franse, Maien S. M. Sachisthal, Maartje E. J. Raijmakers

**Affiliations:** ^1^Department of Psychology, University of Amsterdam, Amsterdam, Netherlands; ^2^NEMO Science Museum, Amsterdam, Netherlands; ^3^Educational Studies and Learn, Free University, Amsterdam, Netherlands

**Keywords:** informal STEM learning, individual interest, wicked problems, network analysis, visitor studies

## Abstract

Science centers and science museums have an important social role in engaging people with science and technology relevant for complex societal problems—so called wicked problems. We used the case of personalized medicine to illustrate a methodology that can be used to inform the development of exhibitions on such wicked problems. The methodology that is presented is grounded in dynamic theories of interest development that define interest as a multidimensional construct involving knowledge, behavior (personal and general) value, self-efficacy, and emotion. The methodology uses a mixed method design that is able to (1) study the predictive effects of background variables on interest, (2) study the interest dimensions predicting individual interest, and (3) identify the most influential interest dimensions. We set up focus groups (*N* = 16, age = 20–74, low SES) to design a survey study (*N* = 341, age 19–89 years olds with a broad range of SES) about people’s interest in personalized medicine. Results of a network analysis of the survey data show that despite the variety in emotions and knowledge about subtopics, these dimensions do not play a central role in the multidimensional interest construct. In contrast, general value and behavior (related to understanding scientific research) seem to be interesting candidates for eliciting situational interest that could have an effect on the more long term individual interest. These results are specific for the case of personalized medicine. We discuss ways in which results of studies with the presented methodology might be useful for exhibition development.

## 1. Introduction

Wicked problems, such as digitization, climate change or future healthcare, do not have clear solutions, but a variety of insights into what the core of the problem is and what the directions are in which solutions should be sought. Multiple perspectives, including a scientific perspective, are relevant for understanding wicked problems. Science centers and science museums have an important social role in engaging people with science and technology relevant to these complex societal problems (Rittel and Webber, 1973; Dillon, 2017).

The development of interest for topics, such as wicked problems, are believed to have multiple phases. The main subdivision made is in situational and individual interest (e.g., Hidi and Renninger, 2006; Ainley and Ainley, 2015), but also three stages have been distinguished (Krapp, 2002b). Situational interest “describes a short term psychological state that involves focused attention, increased cognitive functioning, persistence, enjoyment or affective involvement, and curiosity” (Schiefele, 2009, p. 198). Situational interest needs an external trigger to arise and lasts relatively shortly, for instance for the time span of the activity or the learning event. Individual interest, on the other hand, “refers to a long-term disposition to engage with a particular practice or set of activities” (Azevedo, 2018, p. 110). Someone with an individual interest in a topic engages with the topic without needing external support. The general assumption of this multiple phase model is that a repeated triggering of situational interest precedes the development of a more sustained and ultimately an individual interest. Individual interest is not only thought to be relatively stable, but is also marked by (positive) affect toward the topic, by an increased knowledge about and valuing of the topic (Hidi et al., 2004; Schiefele, 2009). That is, an interest in a topic involves multiple dimensions of cognition, behavior and affect, which form a complex, dynamic interplay (Sachisthal et al., 2019). Retrospective interviews with individuals with an interest in nature point to the importance of overlapping knowledge, skills and identities in the development of an individual interest (Hecht et al., 2019). For science, the importance of the family in shaping students’ engagement, aspirations and achievement, and thus their science interest was well-established (Archer et al., 2012). Archer et al. (2012) explain the complex process of developing an interest in science by the Bourdieusian framework. It is the interplay between the family science capital, the social, economic, and cultural resources that facilitate science achievements, and the habitus, the values, sense of identity, and practices of the family, that shape the development of an interest. Hence, formal education is expected to be just one step in the development of an individual interest in a particular subject, especially for wicked problems involving also a scientific perspective. And thus, informal STEM experiences may importantly contribute to the development of an individual interest. Some museum programs have designed interventions aimed at developing an individual interest in science by intervening on multiple dimensions simultaneously, such as the program of Habig and Gupta (2021) at American Museum of Natural History (New York, United States). In general, however, museums and other informal learning institutions typically contribute to interest development by offering a context for learning about a topic that is joyful, that asks for cognitive engagement and is driven by curiosity (Hecht et al., 2019), that is by triggering situational interest.

How can science museums decide which interest dimensions, including (personal and general) values, emotions, self-efficacy, behavior, and knowledge (Sachisthal et al., 2019) should be highlighted in the exhibition to aid the development of individual interest? Having a focus on complex problems here, first it is important to take into account that interest dimensions may differ across subtopics (Betten et al., 2018) and that opinions may be shaped by background variables such as socioeconomic status (SES), education, and local neighborhood (Bouma et al., 2020). With the goal of identifying dimensions that play an important role within individual interest, we draw on a psychometric network perspective, which allows for the study of psychological constructs such as attitudes (Dalege et al., 2016), and individual science interest (Sachisthal et al., 2019, 2020). In a network of individual interest in a wicked problem, the different interest dimensions need to be included, but so do subtopics of the wicked problem in question (Betten et al., 2018). The dimension lying at the heart of the individual interest can then be identified—which may provide insights into possible leads that can be used to develop exhibitions on the wicked problem.

The aim of the current study is to develop a methodology for science museums to identify topic-specific leads to improve the context for triggering situational interest that could contribute to the development of an individual interest of a diverse population. The involvement of people of all ages, backgrounds and stages of life is an important goal for museums (Falk and Dierking, 2019). The design of the exhibition to trigger the situational interest of a variety of people is a first step in that direction. The methodology we follow adheres to a dynamic conception of interest (Ainley, 2017), where interest can be seen as a network of nodes that represent subtopic-specific dimensions (e.g., values, beliefs, emotions, knowledge, and behavior) that mutually influence each other (Sachisthal et al., 2019). The structure of a psychometric network is given by the interactions of the nodes, that is, the partial correlations of pairs of nodes corrected for all other nodes. The so-called central node has the greatest impact on the network as a whole. Central nodes may be effective targets for interventions (Borsboom and Cramer, 2013; McNally, 2016; Zwicker et al., 2020), in our case to stimulate individual interest development. With the same reasoning, nodes that are not well connected to the rest of the network are not expected to be effective subtopic-specific dimensions to contribute to the development of individual interest, not saying that they could not elicit a situational interest. For example, Sachisthal et al. (2019) show that locus of control and self-reported pro-environmental behavior were the most central constructs of interest within a network of climate change beliefs and interest. The results of the current study will give science museums (e.g., content specialists and exhibition developers) a better understanding of how to trigger the interest of a diverse group of adults in a specific wicked problem, that is personalized medicine.

We studied people’s interest in personalized medicine to illustrate the methodology described above. This topic will be part of an exhibition about scientific innovations in healthcare that has been developed and programmed by NEMO Science Museum in 2022–2023. Personalized medicine is an umbrella term covering medical models (e.g., Precision medicine and P4 medicine) in which prevention, diagnosis and treatment are aligned with patients’ specific needs (Pokorska-Bocci et al., 2014). The aim is to treat patients earlier and select therapies that are accurate and effective, aligned with the patient’s individual profile (Pokorska-Bocci et al., 2014; Budin-Ljøsne and Harris, 2016). To that end, it uses genetic, clinical, environmental and lifestyle information about the patient. Personalized medicine is a social issue that matters to all citizens in society. Sooner or later everyone will have to deal with health issues, and changes in health care will impact the whole society.

### 1.1. Research questions

1. What background variables, including socioeconomic background, are predictors of participants’ interest in personalized medicine? (RQ-1).
2. Which interest dimensions best predict participants’ interest in personalized medicine? (RQ-2).
3. What is the central interest dimension that has the greatest impact on the overall interest in personalized medicine? (RQ-3).

### 1.2. Current studies

In order to answer the research questions, we used a mixed methods approach. First, groups of adults from a low SES participated in focus groups. This sample was chosen to make sure the later developed survey would be suitable for a broad audience, which was needed to answer RQ-1. The developed survey was distributed to a broad sample and was used to answer the three research questions. Different analyses strategies were used to answer the research questions, with RQ-1 and RQ-2 being answered using regression analyses and RQ-3 being answered using a psychometric network approach.

## 2. Materials and methods

### 2.1. Focus groups

The focus groups served as a first study to explore how to survey a broad audience, including adults who do not naturally visit science museums, about their interest in personalized medicine. A focus group is “a carefully planned discussion designed to obtain perceptions on a defined area of interest in a permissive, non-threatening environment” (Krueger, 1994). This qualitative research method can offer insight into sources of complex behaviors and motivations. Because participants question each other and explain their own point of view to others, it yields more than separate individual interviews (Evers, 2015). The focus group allows the researcher to study the ways in which individuals collectively understand a phenomenon and construct meaning around it (Bryman, 2016, p. 502).

The sessions were designed to invite adults of low SES to engage in conversation about personalized medicine. Personalized medicine relates to several complex issues. Through community conversation gatherings in which experts in the field or community members were asked to share their concerns around personalized medicine, the Boston Museum of Science and Tufts Clinical and Translational Science Institute (2021) identified common themes and areas of focus. We used these themes as a starting point to design the focus groups, in which we adhered to the structure of P4 medicine: Predictive, preventive, personalized and participatory (Hood, 2008).

#### 2.1.1. Participants

Participants with lower SES were recruited through an external agency^1^ selecting adults with an educational level of secondary vocational education or below and a gross annual income below modal in three age ranges. The participants did not know in advance what topic the study was about. A total of 24 participants signed up, eighth for each age group. A total of 18 participants showed up*, consented to participate in the study and completed the focus group. [*Note that more than half of the 18–30 year olds did not show up, while the 50+ group had one participant too many]. In the final sample the average age was 50 years (SD = 16). Group 1: *N* = 3; Age range 18–30; 1 male, 2 female; 1 secondary education, 2 vocational education. Group 2: *N* = 6; Age range 30–50; 3 males, 3 females; 1 primary education, 2 secondary education, 3 vocational education. Group 3: *N* = 9; Age range 50 and older; 5 male, 4 females; 5 secondary education, 4 vocational education.

#### 2.1.2. Procedure

Prior to the session, participants received an information letter and signed a consent form confirming that they (1) were 16 years of age or older, (2) had read and understand the information, (3) agreed to participate in the study and to the use of the obtained data, (4) reserve the right to withdraw this consent without giving any reason, and (5) reserve the right to stop participating in the study at any time. The focus group had an established structure, which included five parts: introduction round, introduction to the topic of personalized medicine, small-group discussion using a worksheet, group discussion using statements, and brainstorming on exhibition ideas. Two facilitators were present during the sessions: a scientist-practitioner who guided most of the session and conversations and a student who took care of the audio recordings. The focus groups lasted 2 h. Afterward, participants received 20 euros from the recruitment agency.

#### 2.1.3. Materials

The focus group consisted mainly of verbal activities. To give the physically minded participants a pleasant start we began the session with a hands-on icebreaker activity (Broerse et al., 2014). Using a small bag with Lego bricks, participants were asked to build a duck. Some participants finished quickly; others found the task difficult. Then each participant introduced themselves and told a little anecdote about a duck. Although everyone had been given the same building blocks, different variations of ducks emerged—making a parallel with the purpose of the focus group: the conversations today are not about who makes the best or smartest comments, we are interested in all your ideas (Broerse et al., 2014).

The topic of personalized medicine was introduced using the example of asthma (UC San Francisco (UCSF), 2015). Participants were presented with three situations in which a fictional protagonist had an important choice to make, related to personalized medicine (e.g., this lady is often short of breath. Someone in her family died young from lung problems. She has read that people with lung problems can participate in a heredity test. She faces an important decision: is she going to apply for a hereditary test or not? How does she make her choice?). In pairs, participants discussed the situation outlined. A worksheet stimulated them to name cognitive content (what questions come to mind?), affective involvement (what emotions come to mind?) and relevant behaviors (what actions do you take?). To stimulate discussion, the groups changed composition before a new situation was introduced. After a short break, participants discussed in a plenary session three statements related to personalized medicine (e.g., The responsibility for decisions about healthcare lies with the doctor, not the patient). As a concluding activity, participants were asked what they would like to see or experience as a visitor to a scientific innovations in healthcare exhibition.

#### 2.1.4. Analysis strategy

The focus group discussions were recorded and transcribed afterward. A qualitative analysis was performed by directive content analysis (Hsieh and Shannon, 2005) where the participants’ statements were categorized into the six interest dimensions (Rotgans, 2015; Budin-Ljøsne and Harris, 2016; Sachisthal et al., 2019): Knowledge, behavior, emotion, self-efficacy, personal value, and general value. 15–20% of the data was double-coded, the inter-observer reliability was found to be “moderate” (Landis and Koch, 1977): percentage agreement = 82, and kappa = 0.53.

### 2.2. Online survey

#### 2.2.1. Participants

Participants with lower and higher SES were recruited through an external agency (see text footnote 1) selecting adults with an educational level of secondary vocational education or below and a gross annual income below modal and adults with an educational level of higher vocational education or higher and a gross annual income above modal. The participants did not know in advance what topic the study was about. A total of 518 participants signed up of which 360 consented to participate in the study and completed the survey to the end. A total of 19 participants were excluded from analysis, 11 for providing repetitive answers, 5 for completing the survey too quickly (< 5 min), and 3 for whom both applied. In the final sample (*N* = 341), the mean age was 49.81 years (*SD* = 15.61). A total of 180 participants identified themselves as male, 157 as female and 4 as other/ I’d rather not say. The educational level (and SES) was low for 173 participants (78 Secondary education, 95 Vocational education) and high for 168 participants (108 Bachelor, 60 Graduate).

#### 2.2.2. Procedure

Participants completed the online survey at home. After actively consenting to participate in the study (see section 2.1.2), participants were first asked to provide information on background measurements (e.g., age, gender, and zip code). This was followed by a short animation (2 min) to introduce the topic of personalized medicine. Then the questions on the 6 subtopics were offered. The total duration of participation was 15 min. After completing the survey, participants received 5 euros from the recruitment agency. Participants cannot be traced from the background data.

#### 2.2.3. Materials

Based on results of the focus groups wicked problem questionnaires were constructed consisting of five subtopics related to personalized medicine: (1) Future health, (2) Adapt lifestyle to stay healthy, (3) Having a say in medical decisions, (4) Share medical data to improve healthcare, (5) Participate in scientific research to improve healthcare. As a sixth subtopic, the working title of the scientific innovations in healthcare exhibition was added: (6) How do I live to be 200?. For each subtopic participants answered 7 items on a 5-point Likert scale. Interest in a subtopic was asked both globally (e.g., I find participation in medical decisions an interesting topic, henceforth, global interest) and focused on the six interest dimensions (e.g., I experience a lot of emotions when thinking about my responsibility for medical decisions). A reliability analysis showed that the questionnaires were internally consistent for all 6 subtopics, with Cronbach’s alpha scores between 0.759 and 0.815. In addition, participants were asked two open-ended questions about what they would like to see in a scientific innovations in healthcare exhibition.

The online survey also included questions about participant’s age, gender and educational level, and five more background variables (also see Table 1):

**Table 1:** Factors and covariates, describing eight background variables.

			Total	Lower SES	Higher SES
Ag		M (SD)	49.81 (15.61)	52.50 (15.27)	47.04 (15.51)
Ge	Male	n (%)	180 (53)	80 (46)	100 (60)
	Female	n (%)	157 (46)	90 (52)	67 (40)
	Other	n (%)	4 (1)	3 (2)	1 (1)
Ed	Low	n (%)	173 (51)	173 (100)	0 (0)
	High	n (%)	168 (49)	0 (0)	168 (100)
Se		M (SD)	57.71 (19.04)	50.87 (18.27)	64.76 (17.19)
Ur		M (SD)	2.09^a,b^ (1.83)	1.75 ^a,b^ (1.37)	2.43 ^a,b^ (2.15)
In		M (SD)	3.38 (1.36)	3.72 (1.32)	3.02 (1.31)
Af		n (%)	107 ^a^ (31)	64 ^a^ (37)	43 (26)
He		M (SD)	2.29 (0.84)	2.62 (0.89)	1.95 (0.62)

Total number (n) and percentages (%) of participants’ gender, affinity for the health sector and educational level are presented, and average values (M) and standard deviations (SD) of the other background variables. Ag, age; Ge, gender; Ed, educational level; Se, self-reported socioeconomic status; Ur, degree of urbanization, the average amount of addresses per km^2^ of participants’ residential area; In, individual science interest; Af, affinity for the health sector; He, general health. Total, participants in final sample (*n* = 341); Lower SES, participants with an educational level of vocational education or below and a gross annual income below modal (*n* = 173); Higher SES, participants with an educational level of higher vocational education or higher and a gross annual income above modal (*n* = 168). ^a^Data points were missing for urbanization and affinity for the health sector. Therefore urbanization n_Total_ = 336, n_LowerSES_ = 170, and n_HighSES_ = 166 and affinity for the health sector n_Total_ = 340 and n_LowerSES_ = 172. ^b^Numbers must be multiplied by a thousand.

##### 2.2.3.1. Self-reported socioeconomic status

On a scale of 1 (little money and/or education) to 10 (a lot of money and/or education), participants indicated their socioeconomic background (MacArthur Scale of Subjective Social Status; Adler et al., 2000). Self-reported SES (range 1–10) was used in analysis.

##### 2.2.3.2. Urbanization

A measure of the concentration of human activities based on address density (Dulk et al., 1992), with five categories (low: <500 addresses/km^2^; moderate low: 500–1,000 addresses/km^2^; medium: 1,000–1,500 addresses/km^2^; moderate high: 1,500–2,500 addresses/km^2^; high: > 2,500 addresses/km^2^). Participants’ average address densities (AOD) were retrieved using the zip code digits of their residential areas (CBS, 2020) and were used in analyses.

##### 2.2.3.3. Individual science interest

Participants’ individual science interest (the questions were not topic specific) was assessed by three 5-point Likert-scale questions (α = 0.66), relating to the frequency (e.g., 1 = weekly to 5 = never) with which participants read (online) science-related newspaper articles, visited science museums and listened to or watched science shows on radio, television or the internet. Average interest in science (range 1–5) was used in analyses.

##### 2.2.3.4. Affinity for the health sector

Out of 10 sectors, participants were asked to choose the sector with which they have the most affinity. Affinity for the Health sector (dichotomous variable) was used in analyses.

##### 2.2.3.5. General health

On a scale of 1 (good) to 5 (bad), participants indicated their general health. Self-reported health (range 1–5) was used in analysis.

#### 2.2.4. Analysis strategy

To study what background variables are predicting participants’ interest in the personalized medicine related subtopics and the exhibition’s working title (RQ-1), a MANCOVA will be performed with the average interest in the six subtopics as dependent variables, and the eight background variables as factors and covariates.

To study which interest dimensions best predict participants’ global interest in personalized medicine related subtopics and the exhibition’s working title (RQ-2), six Backward regressions will be performed with global interest in a subtopic as a dependent variable and the six interest dimensions of a subtopic as predictors.

To infer the central interest dimensions of personalized medicine related subtopics (RQ-3), a psychometric network approach was used (*cf.*, Sachisthal et al., 2019). In psychometric networks the included measures (i.e., the items) are represented by so-called *nodes*, and their relations are represented by *edges*, which show direct connections between nodes, after controlling for all other nodes within the network (i.e., partial correlations; Epskamp et al., 2012; Schmittmann et al., 2013). Each questionnaire item on personalized medicine is represented by a node, meaning that the network model includes 30 nodes in total, based on six interest dimensions across five subtopics. We did not include the sixth subtopic. How do I live to be 200, because this subtopic was formulated from the perspective of the exhibition instead of interest in personalized medicine. To estimate the network, the *estimateNetworks* function embedded within the *R*-package *bootnet* was used (Epskamp et al., 2012). *Mgm* (mixed graphical modeling; Haslbeck and Waldorp, 2020) was used as the default to estimate the network given that it has been shown to discover true edges when the sample size is small (Isvoranu and Epskamp, 2021). Mgm can be used when variables differ in scale (i.e., binary and continuous data; Haslbeck and Waldorp, 2020). Edges are estimated using general linear models that are penalized on a node-wise basis. This is done by firstly predicting each node by all other nodes using a regularized general linear regression. In the second step, all estimated regression weights are then combined and averaged into the resulting network model. Model selection was done based on cross-validation (CV) prediction accuracy, as this form of model selection performed best in small sample sizes when the goal was to discover true edges (Isvoranu and Epskamp, 2021). A total of 10 cross-validation folds were used (Isvoranu and Epskamp, 2021).

Two network characteristics of the resulting network were investigated: First, we investigated whether communities (i.e., clusters) of nodes formed within the network. This was done using the walktrap algorithm (Pons and Latapy, 2006) which is embedded within the *igraph* package (Csardi and Nepusz, 2006). Communities are groups of nodes that are strongly interconnected. Secondly, we investigated node centrality within the network. More specifically, we determined the strength centrality (i.e., the direct influence one node has on other directly connected nodes), which is the most stable measure of node centrality (Epskamp et al., 2018; Isvoranu and Epskamp, 2021). Strength centrality is computed by summing up the absolute values of edges a given node has (Opsahl et al., 2010). The function *centralotyPlot* included in the *R*-package Qgraph was used to plot centrality (Epskamp et al., 2012).

Lastly, we tested the stability and accuracy of the network and node centrality using bootstrap and edge difference tests implemented in the *R-package bootnet* (Epskamp et al., 2018). In the context of psychometric networks, accuracy refers to the degree to which the network structure stays the same given sampling variation. The stability of node centrality refers to whether the interpretation of node centrality (i.e., which node is the most central?) stays the same even with less observations. Testing the stability and accuracy of the network and centrality is of importance given the often rather small sample sizes used to estimate psychometric networks. We followed the three steps outlined by Epskamp et al. (2018) to determine the stability and accuracy of the network: (1) edge-weight accuracy is determined based on bootstrapped confidence intervals; (2) stability of node centrality is determined based on centrality of networks estimated in parts of the observations; and (3) testing whether edges (centrality of nodes) differ significantly from other edges (nodes) using bootstrapped difference tests. Please refer to the Supplementary Material for a more thorough description of the analyses done.

## 3. Results

### 3.1. Focus groups

The conversations participants had during the three focus groups are summarized by interest dimension. Only if there were clear differences between the age groups this was indicated for the relevant dimension.

#### 3.1.1. Knowledge

Most participants did not have much prior knowledge of the topic of personalized medicine. However, the topic did prompt many questions for all ages. These were about the example of asthma, how scientific research works and data privacy. In addition, participants were curious about technical innovations. They suggested making the technology of the future visible in the new exhibition on health care. This proposal appeared from all age groups.

#### 3.1.2. Behavior

Two types of behavior were discussed by participants in response to the questions presented. One type was about actions to learn more about a subtopic. When gathering information, the older generation (50+) would consult their inner circle (loved ones/family) and all participants would consult the Internet and a doctor for information about a disease or for making an important medical choice. The other type of behavior was about changing lifestyle where the conversation was often about life choices. Whereas the younger generation (18–50) were not so quick to give up an “unhealthy” lifestyle, the older generation (50+) was willing to do so. Following this, the participants proposed a similar exhibition, in which the consequences of certain life choices would become visible.

#### 3.1.3. Emotions

The topic of personalized medicine evoked both negative and positive emotions among the participants, although negative emotions such as fear, helplessness and stress dominated. These negative emotions were mentioned when the protagonist’s medical situation was discussed, but also when it came to insecurity of medical data. This was true for all three age groups.

#### 3.1.4. Self-efficacy

This was about confidence in one’s own role. Participants were able to reflect on the topic of personalized medicine. Sometimes the conversation was somewhat uncertain at the beginning but this disappeared as the focus group progressed. On the subtopic of medical decisions, many participants agreed: a patient should have a say in medical decisions, but the physician has the responsibility to properly assess a patient’s ability to do so. Participants were also good at indicating whether they themselves needed some form of support in making medical decisions.

#### 3.1.5. Personal value

The personal value examples mentioned by participants dealt with themes such as self-determination, knowing where one stands, growing old healthily, carefree living, quality of life, data security and privacy. It turned out that a majority of the participants wanted to know as much as possible about his/her health in the future, in the interest of preventing disease and making the best choices regarding a possible desire for children. In this regard, a difference in age was seen, however. The younger generation (18–50) gave more value to carefree living while the oldest age group (50+) gave more value to knowing where one stands and growing old healthily.

#### 3.1.6. General value

The general value of the topic of personalized medicine was seen by participants primarily in subtopics such as privacy and data security and mental health. The possible insecurity of medical data was discussed and what consequences this might have in terms of job security or insurance. The younger generation (18–30) also expressed a need for understanding from society about invisible mental health issues, among others. The older generation (50+) responded with a need for understanding from society about other medical conditions such as coughing in public.

### 3.2. Online survey

#### 3.2.1. Descriptions of participants’ background variables

##### 3.2.1.1. Background variables

Five of the eight background variables are described below (see also Table 1), age, gender and educational level are described in section 2.2.1. *Self-reported SES.* On a scale of 1 (low) to 10 (high), participants rated their social economic status (SES) slightly above the average of 5.5 (*M* = 5.771, *SD* = 1.904). *Urbanization.* Participants’ average neighborhood address density (AOD) was 2090 (SD = 1830), indicating that participants’ residential area is on average highly urban. *General science interest*. On a scale of 1 (weekly) to 5 (never), participants were not frequently seeking scientific information through the media (*M* = 3.38, *SD* = 1.36). Two-thirds (67%) of the participants sometimes (weekly to annually) watch or listen to a science program on TV/radio or the Internet. More than half of the participants (57%) never visit science museums and half of the participants (50%) never read the science supplement of an (internet) newspaper. *Affinity for the health sector.* One-third of participants (31%) indicated an affinity for the Health and Wellness sector when asked to choose from 10 sectors. *General health.* Two-thirds (67%) of participants rated their health as (very) good. On a scale of 1 (good) to 5 (bad), participants rated their health below the average of 3 (*M* = 2.29, *SD* = 0.84).

##### 3.2.1.2. Lower socioeconomic status versus higher socioeconomic status

Participants with lower SES (*N* = 173) were almost 3 years older and rated their socioeconomic background 15 points lower (Age, *M* = 52.50, *SD* = 15.28; Self-reported SES, *M* = 50.87, *SD* = 18.27) than participants with higher SES (*N* = 168; Age, *M* = 47.04, *SD* = 15.51; Self-reported SES, *M* = 64.76, *SD* = 17.19.). The percentual gender distribution (male; female; other) differed between the lower (46; 52; 2) and higher (60; 40; 1) SES groups. Science information was less often read, visited, watched or listened to in the lower (*M* = 3.72, *SD* = 1.32) than in the higher (*M* = 3.02, *SD* = 1.31) SES group. While the affinity for the health sector was higher in the lower SES group (37%) than the higher SES group (25%). Participants with lower SES rated their health less often (40%) as (very) good than participants with higher SES (60%).

##### 3.2.1.3. Correlations between background variables

Spearman’s rho shows a medium to strong correlation (rho = −0.403, *p* < 0.001) between participants’ educational level and general health: higher education and better health are related. Spearman’s rho shows a medium to strong correlation between participants’ educational level and self-rated SES (rho = 0.368, *p* < 0.001): higher education and higher socioeconomic background are related. Spearman’s rho shows a weak to medium correlation between participants’ educational level and individual science interest (rho = −0.282, *p* < 0.001): higher education and frequently seeking scientific information through the media are related. Spearman’s rho shows a medium to strong correlation between participants’ general health and self-rated SES (rho = −0.318, *p* < 0.001): better health and higher socioeconomic background are related. Spearman’s rho shows a weak to medium correlation between participants’ health and age (0.200, *p* < 0.001): better health and younger age are related.

#### 3.2.2. Descriptions of participants’ interest in personalized medicine

On average, participants (*N* = 341) were interested in the five personalized medicine related subtopics: Future health (*M* = 3.744, *SD* = 0.572), Adapt lifestyle to stay healthy (*M* = 3.748, *SD* = 0.557), Having a say in medical decisions (*M* = 3.710, *SD* = 0.603), Share medical data to improve healthcare (*M* = 3.737, *SD* = 0.586) and Participate in scientific research to improve healthcare (*M* = 3.631, *SD* = 0.597). Participants had no interest in the exhibition’s working title How do I live to be 200? (*M* = 2.747, *SD* = 0.836). Their interest in this subtopic was significantly lower than the five personalized medicine related subtopics [*F*(5, 1700) = 252.125, *p* < 0.001, *eta^2^* = 0.426]. See Table 2.

**Table 2:** Participants’ interest in personalized medicine.

	GI	K	B	E	S	P	G	Total-i
	M (SD)	M (SD)	M (SD)	M (SD)	M (SD)	M (SD)	M (SD)	M (SD)
FH	4.13 (0.96)	2.87 (0.84)	4.08 (0.89)	2.87 (1.05)	4.04 (0.78)	4.07 (0.82)	4.15 (0.81)	3.74 (0.57)
AL	4.08 (0.81)	3.50 (0.87)	3.76 (0.86)	2.86 (1.02)	4.08 (0.76)	4.00 (0.81)	3.97 (0.90)	3.75 (0.56)
SM	4.26 (0.84)	2.93 (1.00)	3.86 (0.89)	2.92 (1.09)	4.01 (0.83)	4.10 (0.85)	3.89 (0.88)	3.71 (0.60)
SD	4.05 (0.88)	3.00 (1.00)	4.00 (0.89)	2.74 (1.07)	4.19 (0.78)	4.13 (0.89)	4.05 (0.88)	3.74 (0.59)
PR	4.09 (0.91)	2.89 (1.06)	4.00 (0.88)	2.67 (1.03)	4.03 (0.80)	3.76 (0.96)	3.99 (0.87)	3.63 (0.60)
HL*	2.80 (1.34)	2.02 (0.99)	3.13 (1.37)	2.62 (1.16)	3.33 (1.16)	2.67 (1.22)	2.65 (1.24)	2.75 (0.84)
Total-s	3.90 (0.62)	2.87 (0.67)	3.80 (0.66)	2.78 (0.84)	3.95 (0.60)	3.79 (0.56)	3.78 (0.65)	

Mean values (M) and standard deviations (SD) of participants’ interest in personalized medicine related subtopics and the exhibition’s working title are presented (*N* = 341). FH, future health; AL, adapt lifestyle to stay healthy; SM, having a say in medical decisions; SD, share medical data to improve healthcare; PR, participate in scientific research to improve healthcare; HL, how do I live to be 200?. Interest in a subtopic was asked globally (e.g., I find participation in medical decisions an interesting topic) and focused on the six interest dimensions (e.g., I experience a lot of emotions when thinking about my responsibility for medical decisions). GI, global interest in a subtopic; K, knowledge; B, behavior; E, emotion; S, self-efficacy; P, personal value; G, general value; Total-i, the mean value of all 7 items (GI through G); Total-s, the mean value of all 6 subtopics (FH through HL). The exhibition related subtopic is marked with an asterisk.

#### 3.2.3. What background variables, including socioeconomic background, are predicting participants’ interest in personalized medicine? (RQ-1)

To study what background variables are predicting participants’ interest in personalized medicine related subtopics and the exhibition’s working title, a MANCOVA was performed with the average interest in the six subtopics as dependent variables, and the eight background variables as factors and covariates. Because of violation of the covariance matrices Pillai’s Trace statistics were reported.

Using Pillai’s trace, there were non-significant effects of urbanization, educational level and self-reported SES and significant effects of age [*V* = 0.057, *F*(6, 315) = 3.199, *p* < 0.005, *eta^2^* = 0.057], gender [*V* = 0.088, *F*(6, 315) = 5.086, *p* < 0.001, *eta^2^* = 0.088], individual science interest [*V* = 0.106, *F*(6, 315) = 6.208, *p* < 0.001, *eta^2^* = 0.106], affinity for the health sector [*V* = 0.040, *F*(6, 315) = 2.173, *p* < 0.05, *eta^2^* = 0.040] and general health [*V* = 0.047, *F*(6, 315) = 2.601, *p* < 0.05, *eta^2^* = 0.047] on participant’s average interest in the six subtopics.

**Figure 1:**
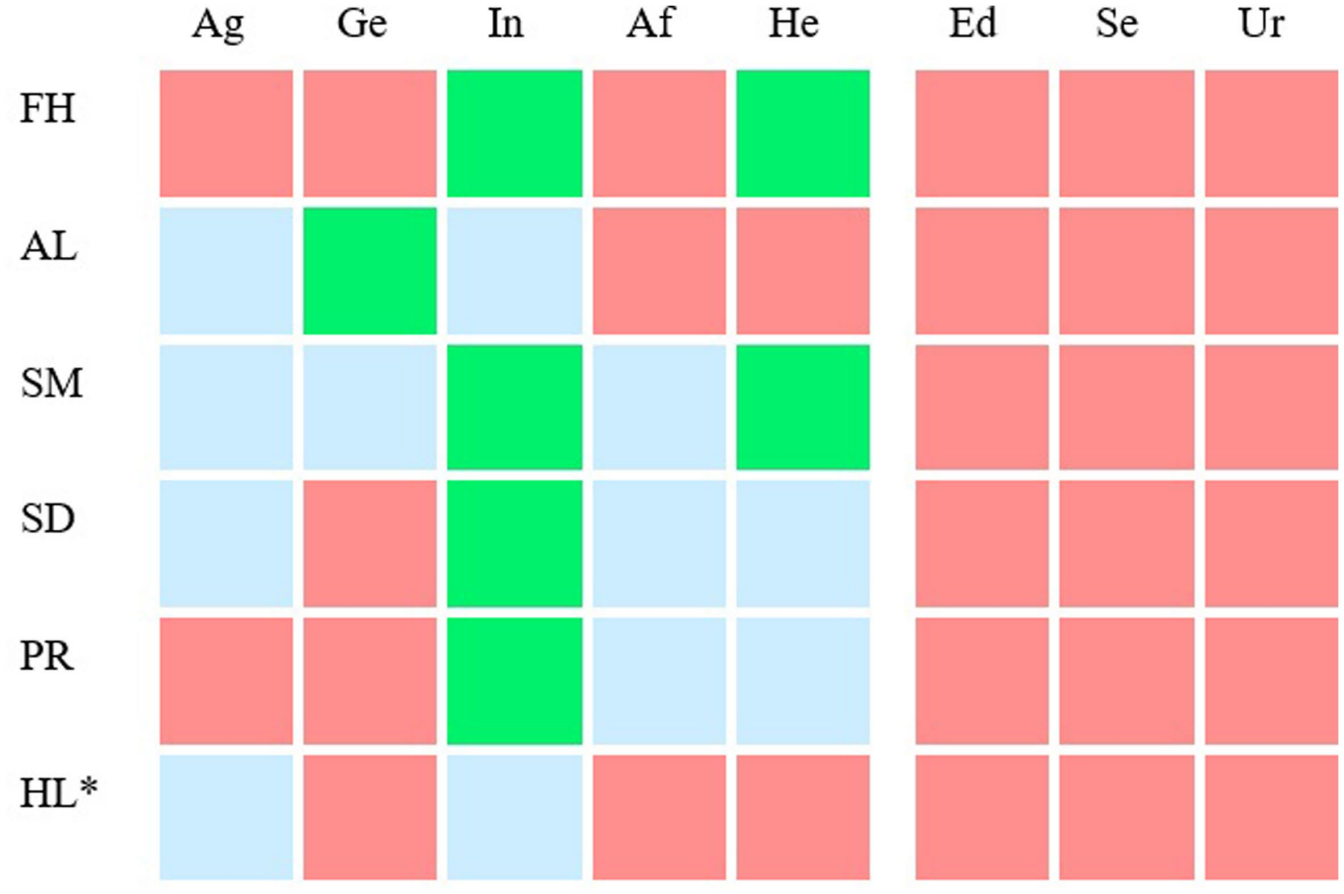
Background variables that predict participants’ interest in personalized medicine related subtopics and the exhibition’s working title. For six subtopics (lines 1–6) the relationship between average interest in a subtopic and eight background variables (column 1–8) was visualized. FH, future health; AL, adapt lifestyle to stay healthy; SM, having a say in medical decisions; SD, share medical data to improve healthcare; PR, participate in scientific research to improve healthcare; HL, how do I live to be 200?; Ag, age; Ge, gender; In, individual science interest; Af, affinity for the health sector; He, general health; Ed, educational level; Se, self-reported SES; Ur, urbanization of the residential area. The color of the cells depict the background variables that do (green and blue) and do not (red) significantly predict participants’ average interest in a subtopic (*p* < 0.001). Note that the effect sizes were small. The effect sizes (*eta^2*) of the green cells are between 0.1 and 0.03 and of the light blue cells between 0.03 and 0.01. The exhibition related subtopic is marked with an asterisk (line 6).

However, separate univariate ANOVAs on the outcome variables revealed non-significant effects of the five background variables on some subtopics (also see Figure 1).

##### 3.2.3.1. Age

Older participants showed more interest in the subtopics Adapt lifestyle to stay healthy, *F*(1, 320) = 4.376, *p* < 0.05, *eta^2^* = 0.013, having a say in medical decisions, *F*(1, 320) = 8.660, *p* < 0.01, *eta^2^* = 0.026 and share medical data to improve healthcare, *F*(1, 320) = 5.777, *p* < 0.05, *eta^2^* = 0.018. than younger participants. However, younger participants showed more interest in the subtopic how do I live to be 200? than older participants, *F*(1, 320) = 4.218, *p* < 0.05, *eta^2* = 0.013.

##### 3.2.3.2. Gender

Females showed more interest in the subtopics Adapt lifestyle to stay healthy, *F*(1, 320) = 11.480, *p* < 001, *eta^2* = 0.035 and having a say in medical decisions, *F*(1, 320) = 6.079, *p* < 0.05, *eta^2* = 0.019 than males.

##### 3.2.3.3. General science interest

Participants who accessed scientific information more frequently were more interested in the subtopics future health, *F*(1, 320) = 18.517, *p* < 0.001, *eta^2* = 0.055, adapt lifestyle to stay healthy, *F*(1, 320) = 9.051, *p* < 0.005, *eta^2* = 0.028, having a say in medical decisions, *F*(1, 320) = 32.305, *p* < 0.001, *eta^2* = 0.092, share medical data to improve healthcare, *F*(1, 320) = 19.804, *p* < 0.001, *eta^2* = 0.058, participate in scientific research to improve healthcare, *F*(1, 320) = 23.323, *p* < 0.001, e*ta^2* = 0.068, and how do I live to be 200?, *F*(1, 320) = 4.369, *p* < 0.05, *eta^2* = 0.013, than participants who did so less frequently.

##### 3.2.3.4. Affinity for the health sector

Participants involved in the health sector showed more interest in the subtopics Having a say in medical decisions, *F*(1, 320) = 5.900, *p* < 0.05, *eta^2* = 0.018, share medical data to improve healthcare, *F*(1, 320) = 5.154, *p* < 0.05, *eta^2* = 0.016, and Participate in scientific research to improve healthcare, *F*(1, 320) = 8.919, *p* < 0.01, *eta^2* = 0.027 than participants who did not have this affinity.

##### 3.2.3.5. General health

Participants with poorer health were more interested in the subtopics future health, *F*(1, 320) = 10.094, *p* < 0.005, *eta^2* = 0.031, having a say in medical decisions, *F*(1, 320) = 10.301, *p* < 0.001, *eta^2* = 0.031, share medical data to improve healthcare, *F*(1, 320) = 5.049, *p* < 0.05, *eta^2* = 0.016 and participate in scientific research to improve healthcare, *F*(1, 320) = 5.864, *p* < 0.05, *eta^2* = 0.018 than participants with better health.

#### 3.2.4. Which interest dimensions best predict participants’ interest in personalized medicine? (RQ-2)

**Figure 2:**
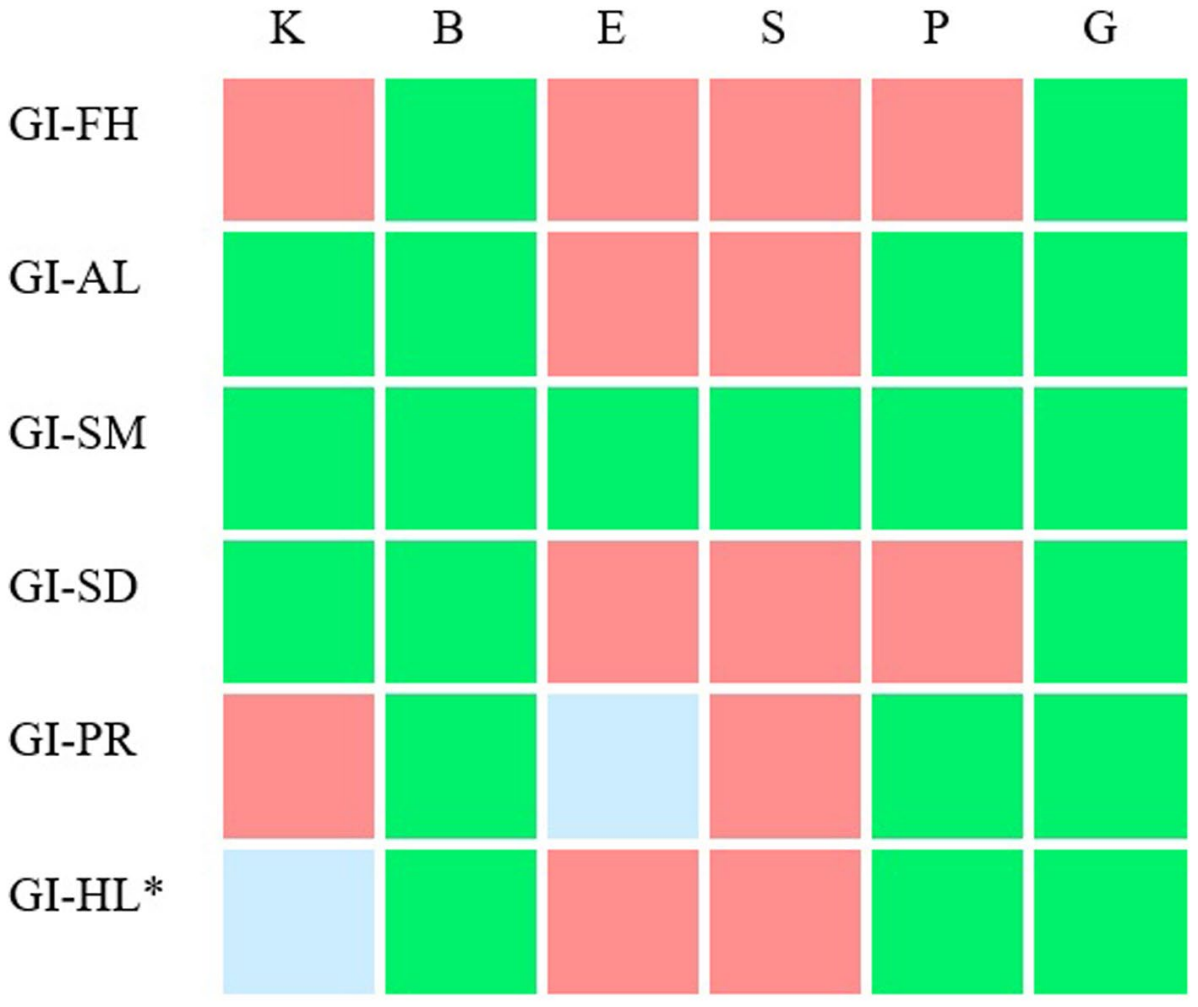
The relation between interest dimensions and participants’ global interest in personalized medicine related subtopics and the exhibition’s working title. For six subtopics, the relationship between participants’ global interest in a subtopic (GI) and interest in the six subtopics (interest dimensions K through G) was visualized. FH, future health; AL, adapt lifestyle to stay healthy; SM, having a say in medical decisions; SD, share medical data to improve healthcare; PR, participate in scientific research to improve healthcare; HL, how do I live to be 200?. The exhibition related subtopic is marked with an asterisk. The color of the cells depict the interest dimensions that do (green and blue) and do not (red) significantly predict participants’ global interest in a subtopic, when performing a Backwards multiple regression with global interest as dependent variable and the six interest dimensions as predictors. Moreover, for the green cells, these interest dimensions contribute significantly and positively to the model. For example: Behavior, emotion, personal value and general value could significantly predict participants’ interest in the subtopic Participate in scientific research to improve healthcare. Behavior, personal value and public interest contributed significantly and positively to this prediction.

To study which interest dimensions best predict participants’ interest in personalized medicine related subtopics and the exhibition’s working title, six Backward regressions were performed with global interest in a subtopic (GI) as dependent variable and the six interest dimensions of a subtopic (K through G) as predictors (a summary of the results is presented in Table 2 and Figure 2).

##### 3.2.4.1. Future health

Interest dimensions behavior (B) and general value (G) could significantly predict participants’ global interest (GI) in Future health, *F*(2, 338) = 141.533, *p* < 0.001, *R^2* = 0.456. Both dimensions contributed significantly and positively to this prediction, *p* < 0.001.

##### 3.2.4.2. Adapt lifestyle to stay healthy

Interest dimensions knowledge (K), behavior (B), personal value (P) and general value (G) could significantly predict participants’ global interest (GI) in Adapt lifestyle to stay healthy, *F*(4, 335) = 86.566, *p* < 0.001, *R^2* = 0.508. All four dimensions contributed significantly and positively to this prediction, *p* < 0.001.

##### 3.2.4.3. Having a say in medical decisions

Interest dimensions knowledge (K), behavior (B), emotion (E), self-efficacy (S), personal value (P) and general value (G) could significantly predict participants’ global interest (GI) in Having a say in medical decisions, *F*(6, 334) = 42.609, *p* < 0.001, *R^2* = 0.434. All six dimensions contributed significantly and positively to this prediction, *p* < 0.05.

##### 3.2.4.4. Share medical data to improve healthcare

Interest dimensions knowledge (K), behavior (B) and general value (G) could significantly predict participants’ global interest (GI) in Share medical data to improve healthcare, *F*(3, 336) = 84.955, *p* < 0.001, *R^2* = 0.431. All three dimensions contributed significantly and positively to this prediction, *p* < 0.001.

##### 3.2.4.5. Participate in scientific research to improve healthcare

Interest dimensions behavior (B), emotion (E), personal value (P) and general value (G) could significantly predict participants’ global interest (GI) in Participate in scientific research to improve healthcare, *F*(4, 336) = 92.957, *p* < 0.001, *R^2* = 0.525. Behavior, personal value and general value contributed significantly and positively to this prediction, *p* < 0.001.

##### 3.2.4.6. How do I live to be 200?

A Backward multiple regression showed that interest dimensions knowledge (K), behavior (B), personal value (P) and general value (G) could significantly predict participants’ global interest (GI) in How do I live to be 200?, *F*(4, 335) = 86.566, *p* < 0.001, *R^2* = 0.508. Behavior, personal value and general value contributed significantly and positively to this prediction, *p* < 0.001.

##### 3.2.4.7. Knowledge

The knowledge question predicts participants’ global interest for the subtopics of adapt lifestyle to stay healthy (AL), having a say in medical decisions (SM), Share medical data to improve healthcare (SD) and How do I live to be 200? (HL).

##### 3.2.4.8. Behavior

The behavior question predicts participants’ global interest for all six subtopics (FH, AL, SM, SD, PR, and HL).

##### 3.2.4.9. Emotion

The emotion question predicts participants’ global interest only for the subtopics of Share medical data to improve healthcare (SD) and Participate in scientific research to improve healthcare (PR).

##### 3.2.4.10. Self-efficacy

The self-efficacy question predicts participants’ global interest for the subtopic of having a say in medical decisions (SM).

##### 3.2.4.11. Personal value

The personal value question predicts participants’ global interest for the subtopics of adapt lifestyle to stay healthy (AL), having a say in medical decisions (SM), participate in scientific research to improve healthcare (PR) and How do I live to be 200? (HL).

##### 3.2.4.12. General value

The general value question predicts participants’ global interest for all six subtopics (FH, AL, SM, SD, PR and HL).

#### 3.2.5. What is the central dimension that has the greatest impact on the overall interest in personalized medicine? (RQ-3)

**Figure 3:**
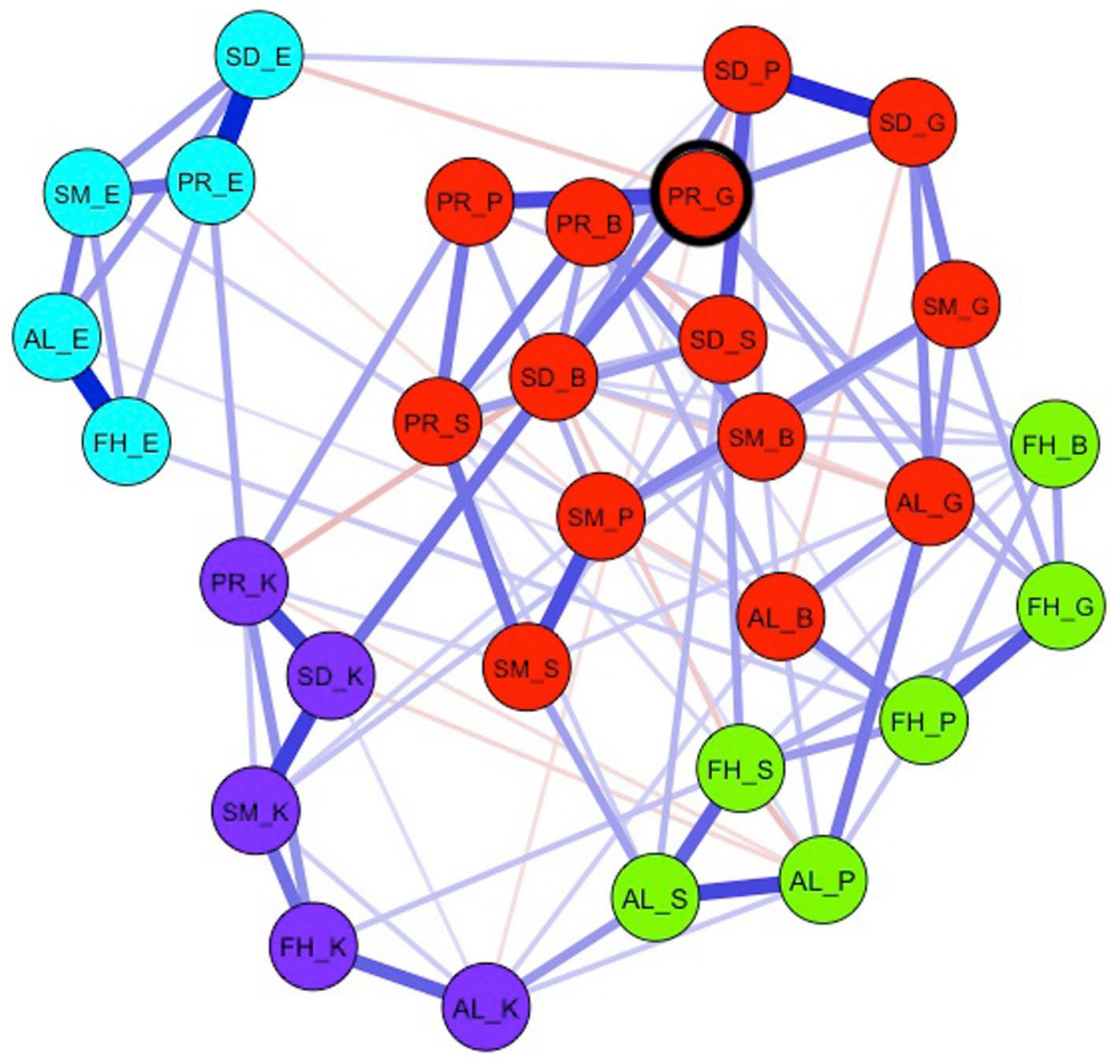
The network model of interest in personalized medicine. FH, future health; AL, adapt lifestyle to stay healthy; SM, having a say in medical decisions; SD, share medical data to improve healthcare; PR, participate in scientific research to improve healthcare; K, knowledge; B, behavior; E, emotion; S, self-efficacy; P, personal value; G, general value. The colors of the nodes correspond to detected communities within the network. Blue (red) edges represent positive (negative) relations between nodes. The most central node (PR_G) is highlighted with a black circle.

The estimated network model of interest in personalized medicine is displayed in Figure 3. The nodes are colored based on their community membership. Visual inspection of the network shows that both the emotion nodes and the perceived knowledge nodes form separate clusters that are relatively sparsely connected with the remaining nodes. These two interest dimensions have thus less influence on the other dimensions. The remaining nodes are relatively closely connected. Most edges are positive, with few, relatively weak negative edges. Please refer to the Supplementary Table S1 for an overview of the (partial) correlations between nodes.

##### 3.2.5.1. Community detection

In total, four different communities were detected in the network. Communities are sets of nodes that have relatively strong connections to nodes within the set compared to nodes outside the set. Two relatively distinct communities represent the clusters of the emotion (green nodes) and knowledge nodes (purple nodes) across the five subtopics. The remaining interest dimension nodes were separated into two relatively closely connected communities. First, one cluster emerged including all but two nodes (i.e., knowledge and emotion) measuring interest in Future health and two nodes adapting one’s lifestyle to stay healthy (i.e., self-efficacy and personal value; yellow nodes). Second, the largest community included all remaining nodes of the subtopics: Having a Say in Medical decisions, Share medical data to improve healthcare, and participate in scientific research to improve healthcare, as well as the general value node and the behavior node of the adapting one’s lifestyle subtopic (red nodes).

##### 3.2.5.2. Node centrality

A network of interacting nodes may show complex dynamics when the activity of one node (i.e., the item score) is changed. The node that has the strongest connections to other nodes is considered to have the strongest impact on other nodes in the network. For the network model of interest in personalized medicine, the node with the highest strength centrality is the node “Finding it important for society to participate in scientific research” (PR_G). This node has the strongest direct connections to other nodes in the network. Hence, this subtopic may be an interesting target for intervention. It must be noted that the stability of the result is not optimal, meaning that the centrality of some nodes is comparable (see Supplementary Material). For the most central node, only one node, “I wonder how large-scale sharing of medical data could change healthcare in the future” (SD_B), has a somewhat comparable strength. Note that both nodes have to do with understanding scientific research.

## 4. Discussion

### 4.1. Getting adults to explore difficult topics that require broad civic engagement

Wicked problems often deal with complex issues that are not easy to solve and at the same time require broad social engagement. Understanding scientific innovations is important for developing a personal perspective on these types of problems. Science centers and science museums play an important social role in engaging people in science and technology relevant to these complex social problems. As a science museum, how can a broad and diverse audience be encouraged to further develop their own perspectives on these complex issues? We design a methodology for science museums that provides starting points for this—with a focus on the development of individual interest.

### 4.2. The case of personalized medicine

During the concept development of an exhibition, the exhibition team (e.g., content specialists and exhibition developers) explore what aspects, questions, concepts and subtopics might be relevant to present in an exhibition on a particular topic (Neves, 2002). One way to select relevant subtopics is from the perspective of science, for example by engaging with scientists to hear what is cutting edge in their field or what are new trends in a particular area of science. Another way to select relevant subtopics is from the perspective of the potential visitor, what do visitors find interesting subtopics and what does this interest look like? In the current study we take a dynamic perspective on the development of people’s individual interest by studying the structure of their interest for personalized medicine. Potential subtopics of interest were identified using a Focus group approach.

#### 4.2.1. Conversations about personalized medicine

In order to survey a diverse adult audience’s interest in the topic of personalized medicine, we first explored whether and how adults who are not likely to visit museums (e.g., adults of low SES; Falk, 2009; Dawson, 2014) think and talk about the topic. During focus group discussions, we observed what knowledge, attitudes and emotions these adults have about and in relation to personalized medicine.

The structured format of the focus groups and the introduction of personalized medicine with a concrete example (asthma) helped participants discuss a multitude of subtopics about Future health and the predictive, preventive, personalized and participatory sides of personalized medicine (Pokorska-Bocci et al., 2014). For example, questions that were discussed included: Do you want to know everything about your health in the future, would you participate in a heredity test, are you willing to change your lifestyle, would you share your healthcare data to improve healthcare, and who is responsible for making decisions about the healthcare process? The use of worksheets ensured that participants also discussed the topics from the different dimensions of interest (Knowledge, Behavior, Emotion, Self-efficacy, Personal, and General Value).

Based on the example used in the focus group (asthma), a short video was made which was used to introduce the topic of personalized medicine to individuals filling in the survey. Moreover, insights gathered in the focus groups were used to design the questionnaires. For instance, examples mentioned by participants during the focus groups were used to design the questionnaire items.

##### 4.2.1.1. Knowledge and behavior

Focus group participants indicated they did not have much prior knowledge about personalized medicine but had many questions. They were also curious about innovations in health care. Regarding actions related to personalized medicine, both what they did or did not do to stay healthy and what they undertook to learn about the topics were discussed. In the questionnaires, we included items questioning the degree of knowledge (Knowledge) and the degree of curiosity about the impact of innovations on health care (Behavior). Being based on a group of low SES individuals, we were interested whether knowledge and curiosity in the topic may differ for adults with higher education or for adults who have a lot to do with health care in their daily lives (educational level, self-assessed SES, general health, affinity with health sector).

##### 4.2.1.2. Emotions and self-efficacy

Anxiety, Irritation, Sadness, Anger, Nervousness, Inability, Uncertainty, Joy, Interest, Satisfaction and Relief are examples of emotions and feelings mentioned during the interviews. Because of the multitude of different emotions that the topic of personalized medicine evoked, we decided to only include one question on the degree of experiencing emotions when thinking about the different subtopics. This contrasts with research into interest on other topics: for Climate Change the specific emotions of hope and distress were included (Sachisthal et al., 2019) whereas enjoyment was included in Science interest networks. Both one’s own self-efficacy (e.g., confidence in one’s own ability to make lifestyle changes) and the efficacy of others were discussed. Reduced self-efficacy was mentioned with examples such as “It also has to do with your cognitive ability, how can you get around?” and “People with a language barrier, low literacy, we need to be mindful of that within personalized medicine.” Only one’s own self-efficacy was included in the survey.

##### 4.2.1.3. Personal and general value

Personal and general value examples that were mentioned in the interviews linked to the predictive, preventive, personalized and participatory sides of personalized medicine (Pokorska-Bocci et al., 2014). For example, “knowing where one stands” linked well to participating in scientific research (predictive), “growing old healthily” and “carefree living” to adapting the lifestyle to stay healthy (preventive), “data security” and “privacy” to sharing medical data to improve healthcare (personalized) and “self-determination” to having a say in medical decisions (participatory). “Quality of life” was an example that was discussed in relation to health in the future. We drew on these examples when designing the personal and general value items in the questionnaires. Therefore, these items were more content-related than, for example, the emotion or knowledge items of the questionnaires.

#### 4.2.2. The role of background variables in interest (RQ-1)

To design exhibitions that are accessible to a broad adult audience, it is important to know the diversity of interest in the subject matter in relation to background variables.

Results show that individual science interest is a good predictor of interest in personalized medicine. Participants who more frequently sought scientific information through the media were more interested in personalized medicine than participants who did not. This was true for all six subtopics (including the exhibition working title), although the effect sizes for some subtopics were small. The effects of science interest were greatest for the personalized medicine subtopics of having a say in medical decisions, sharing medical data, and participating in scientific research.

One explanation is that people who are regularly informed about scientific developments through the media are also exposed to health care related topics, and therefore know more about these topics. Another explanation is that people who regularly search for scientific information are generically interested in scientific innovation regardless of the topic, much interest-development literature however, shows that interest is topic-specific (Tsai et al., 2008; Krapp and Prenzel, 2011; Sachisthal et al., 2019).

In addition to individual science interest, participants’ health is related to their interest in the subtopics. In particular, participants with poorer health are more interested in Future health and having a say in medical decisions than participants with better health. One explanation for this may be that participants with poorer health conditions are more likely to deal with these subtopics in daily life and for example have experience with the situation where doctors do or do not involve patients in medical decisions.

Another way that participants may come into contact with the subtopics in everyday life is through their occupation, which we examined through their affinity for the health sector. However, we showed only small effects here, of which affinity with the health sector had the most effect on interest in participating in scientific research. One explanation for the small effects may be that health is a somewhat special topic, since it affects us all.

The informal science education (ISE) literature suggests that people from lower-economic groups are underrepresented in the museum (Falk, 2009; Dawson, 2014). Therefore, in the current study, we measured educational level and gross annual income (high/low SES), urbanization of the residential area, and self-reported SES.

Against our expectation, the survey shows no relationship between these background characteristics and participants’ interest in the main topic of personalized medicine. All subtopics presented in the survey were of interest by both high and low SES adults (and high and low educated).

The choice of subtopics does not seem to be a limiting factor for the inclusive design of an exhibition that interests a wide audience. But it does not alter the fact that there may be other barriers that may cause adults with lower socio-economic backgrounds not to visit an exhibition about scientific innovations in healthcare in the museum after all, such as infrastructure access, literacy and community acceptance (Dawson, 2014). One possible explanation for why education level (SES) does not play a role in interest in the topics is the negative correlation between education level and health. The expectation is that people who are more educated are more interested in the topics. What contradicts this is that the lower educated in the study had poorer health and it appeared that people with poorer health are more interested in the personalized medicine subtopics.

#### 4.2.3. Dimensions of individual interest (RQ-2)

To develop exhibitions that elicit situational interest in a more robust way, it is important to have insight into adults’ individual interest in the subtopics and the nature of this interest.

Adults thought future health, Adapt lifestyle to stay healthy, having a say in medical decisions, Share medical data to improve healthcare and participate in scientific research to improve healthcare were all interesting aspects of personalized medicine. The nature of the interest in all these subtopics was in the general value people saw and in the curiosity about how innovations will impact healthcare (behavior).

General value was also discussed during the focus groups, for example in relation to the subtopic Share medical data to improve healthcare: “But then, who is allowed to see the data? If the boss is allowed to see it, he might start scratching his head as to whether he wants to employ that person. The naturalness disappears, because the boss or employer might see a person [who is genetically predisposed to a certain disease] that [the company] will have to take into account in the future.”

For three subtopics (adapt lifestyle to stay healthy, having a say in medical decisions, and participate in scientific research to improve healthcare), personal value was also related with the interest in the subtopic. Note, that people with poorer health were more interested in the topic of personalized medicine, likely to the importance of the topics to their personal life (i.e., personal value).

In applying the insights to museum practice, it is not that we propose to create programs that only present the general value of scientific innovations in healthcare, or programs that only deal with the potential impact of innovations for the future. Our perspective on interest development is a dynamic one, where we aim to establish positive feedback loops between the different interest dimensions. Therefore, it is important to understand the connections between the different dimensions, which can be done through network analysis.

#### 4.2.4. The interplay between dimensions of individual interest (RQ-3)

We constructed a psychometric network model of interest for personalized medicine by considering interest as a dynamic construct with multiple dimensions (knowledge, behavior, emotion, self-efficacy, personal value and general value). The resulting network shows the interplay between all dimensions for all subtopics. The central node in the network has the strongest direct relationship with the rest of the network and is therefore an interesting entry point to get people generally interested in the main topic. In contrast, the nodes that are more separate from the network can be expected not to contribute to the development of individual interest. Hence, we were able to explore what is the most promising way to generate individual interest for personalized medicine.

For personalized medicine, the central node turned out to be *Finding it important for society to participate in scientific research*. Offering this specific perspective in an exhibition, in combination with other perspectives and subtopics, could play a role in stimulating a more stable, individual interest in the topic of personalized medicine. Centrality indices of other nodes need to be taken with some care since the stability results are not optimal. This result is in concert with findings from the focus groups. Focus group participants felt it was important for society (i.e., general value) to participate in scientific research because by doing so, they help other people and contribute to improving treatments in the future and hopefully reduce costs for society: “If a good medicine is found, you want to contribute to that, it can also enrich your life.,” “I think it is important that everyone is taken seriously, everyone counts,” “It makes me feel good to participate.” Participants also expressed their interest in scientific research, particularly in how it works and what it means to participate: “Who is leading the research?,” “Is the research meaningful?,” “How long would it take?,” “And then what happens to my data?”

In contrast, in the network emotion and knowledge as dimensions of interest appeared to have little or no connection with the other interest dimensions. The fact that the knowledge questions of all subtopics together form a cluster means that this knowledge is domain-general (within the domain of personalized medicine) and that the level of knowledge is not directly related to participants’ interest in the topic. The same is true for emotions. Participants did report experiencing emotions when thinking about the subtopics, but the extent to which they did so was not directly related to their interest. Therefore, showing the emotional dimension of the topic is not expected to facilitate a more stable interest.

Examples of negative emotions expressed by focus group participants when they imagined themselves in the role of the protagonist were: “I would feel nervous, because you do not know what to expect,” “Uncertainty, you do not know what such an examination looks like,” “Sadness, because your body is letting you down,” “Fear, that you cannot breathe properly,” “It gives me an oppressive feeling, when decisions are made for you.” Examples of positive emotions were: “Relief, when the result of a hereditary test is good,” “Happy, when you can help other people by participating in research.”

### 4.3. A dynamic perspective on interest in the museum context

The aim of the current study was to develop a methodology for science museums to identify topic-specific leads to increase engagement of a diverse population. The methodology consisted of 7 steps. Step 1. Selecting subtopics that are relevant to a main topic. Step 2. Translating these subtopics into a concrete context. Step 3. Testing whether the selected subtopics and context prompted a conversation. Step 4. Analyzing the focus group discussions for the 6 dimensions of interest. Step 5. Based on previous steps, constructing a survey for adults with an important background variety (such as, age, gender, education, SES, local neighborhood, interest in science, affinity with the health sector and perceived health,). Step 6. Performing a network analysis to identify the most influential dimensions for engaging in the main topic (*cf.* Dalege et al., 2016; Sachisthal et al., 2019). Step 7: Work with the exhibition team to discuss how research findings can be translated into the science museum context, how results may or may not be interpreted.

The dynamic perspective on interest development advocates initiating a feedback loop between different dimensions of engagement with a topic. Whereas school-based learning may focus more on topic knowledge and value for society, outside school topics may take on more personal value and an affective dimension. Learning outside school is therefore important for the development of an individual interest in a topic. In science centers, for example, a lot of attention is usually paid to playful, enjoyable interactions. That is, associations are made between the knowledge and affective dimensions of topics. Moreover, visits that take place in a family context, which is a context where personal value may be better recognized. However, family visits to a science museum is not for all families part of their practices. Adapting exhibitions and advertising for them to relevant dimensions of interest of a wide audience is one step to facilitate interest development.

### 4.4. Limitations and future studies

This study was designed to represent a wide audience by selecting participants with different SES and educational levels. Nevertheless, it must be noted that all participants wanted to participate in research in the first place. Results on node centrality in the network model of personalized medicine show that understanding the importance of scientific research is central. At first glance, this result might seem trivial. However, it should be noted that the result concerns *individual variation* in understanding the importance of scientific research, and not a high average value. It is not directly clear that the selection of participants affected this variation within the group of participants who were all recruited through the same procedure. In the case of science interest, for example, boys and girls had significantly different average values of several dimensions of science interest, but the network structure and thus the centrality of nodes, did not differ significantly (Sachisthal et al., 2019).

#### 4.4.1. Performing network analysis

Museums can download free software (JASP), which includes options to perform network analyses (Van Doorn et al., 2021). A step-by-step instruction for performing the network analysis in JASP will be written up in a blog post modeled after this blog post^2^ and the interest data from the current study will be made publicly available (see Supplementary Material). This will offer the possibility to practice network analysis with real data.

## 5. Conclusion

Societies face many wicked problems, which require broad social engagement. In their role of engaging individuals in science and technology, science centers and science museums can take a central role in engaging the public with such wicked problems. Through this engagement, individuals are facilitated to develop a personal perspective on these problems. As wicked problems require broad social engagement, science museums strive to encourage a broad and diverse audience to engage with the complex issues at hand. In the current study, we present a methodology aimed at finding leads on how to design exhibitions that elicit situational interest such that visitors’ engagement might contribute to the development of individual interest. This was done using personalized medicine as a case study and is based on theories of interest development. Our aim has been to develop a methodology for science museums that provides insights concerning the interests in specific topics of a broad population and thereby inspire exhibition development to put individual interest of people central in designing exhibitions.

## Data availability statement

The raw data supporting the conclusions of this article will be made available by the authors, without undue reservation.

## Ethics statement

The studies involving human participants were reviewed and approved by Ethics Review Board of the Faculty of Social and Behavioral Sciences, University of Amsterdam, Netherlands. The patients/participants provided their written informed consent to participate in this study.

## Author contributions

RF and MR conceived the ideas and designed the study. RF supervised the data collection. RF, MS, and MR performed the statistical analysis and wrote the manuscript. All authors contributed to manuscript revision, read, and have approved the submitted version.

## Funding

The research of Rooske Franse was funded by a Museum Grant of the Netherlands Organization for Scientific Research (NWO) and NEMO Science Museum.

## Conflict of interest

The authors declare that the research was conducted in the absence of any commercial or financial relationships that could be construed as a potential conflict of interest.

## Publisher’s note

All claims expressed in this article are solely those of the authors and do not necessarily represent those of their affiliated organizations, or those of the publisher, the editors and the reviewers. Any product that may be evaluated in this article, or claim that may be made by its manufacturer, is not guaranteed or endorsed by the publisher.

## Footnotes

^1^ https://norstatgroup.com/

^2^ https://jasp-stats.org/2018/03/20/perform-network-analysis-jasp/
