# Supplementary material for: Presenting wicked problems in a science museum: A methodology to study interest from a dynamic perspective
Source: Front Psychol. 2023 Feb 10;14:1113019. doi: 10.3389/fpsyg.2023.1113019 (PMC9951591; doi:10.3389/fpsyg.2023.1113019)
Supplement: Supplementary file 1 [file Data_Sheet_1.docx]

**Presenting Wicked Problems in a Science Museum:
A Methodology to Study Interest from a Dynamic Perspective**

## **Stability and accuracy of the network model**

**Edge stability.** The accuracy and stability of the network model of interest in personalized medicine was assessed using the *bootnet* function included in the R-package *bootnet* (Epskamp et al., 2017). Edge weight accuracy was estimated by bootstrapping the 95% confidence intervals of each edge weight based on 2000 iterations. The 95% confidence intervals per edge weight are displayed in Figure S1. The overlap among the 95% confidence intervals of edge weights indicated that the edge weights are moderately accurately estimated. Based on the *bootnet* results, it was also tested whether any two edges significantly differed in their weight strength. The results of this difference test for all edges that differed significantly from zero are displayed in Figure S2.

**Strength centrality accuracies.** To calculate strength centrality we used the function centralityPlot (Epskamp et al., 2017 for the network shown in Figure 3 of the main manuscript; see Figure S3 for the node centrality plot). The node with the highest strength centrality is the node *Finding it important for society to participate in scientific research* (PR_G). This node thus has the strongest direct connections to other nodes in the network. This subtopic therefore may be a potential target for intervention. Figure S4 shows the differences between node centralities for all nodes based on bootstrap estimations of strengths (nBoots = 2000 in the function *bootnet*). This shows that the strength of Node PR_G is significantly different from most other nodes.

**Strength stability.** To estimate the stability of strength centrality, a method called subsetting bootstrap with 2000 iterations was used, which is implemented in the *bootnet* function (Epskamp et al., 2017). In subsetting bootstrap, the network is re-estimated numerous times after dropping a certain proportion of participants. The order of the strength centrality (from highest to lowest) is correlated to that of the original network including the full sample. Based on the subsetting bootstrap the so-called centrality stability coefficient (CS-coefficient) can be estimated, which should preferably be above .5 for centrality to be deemed as stable (Epskamp et al., 2017). For a visual representation of the subsetting bootstrap for strength centrality, see Figure S5. The stability of strength centralities was insufficient, with the CS-coefficient being CS = .152. Note that this stability measure concerns the order of node centrality of all nodes. Therefore, we will only interpret the centrality of the node with the strongest connections.

**Figure S1.** Bootstrapped 95% confidence intervals of the edge weights of the interest in personalized medicine network.

*Note****.*** The red line shows the estimated edge weights based on the sample, whereas the bootstrapped (2000) edge weight mean per edge is indicated by the black line. The gray area shows the bootstrapped confidence interval per edge weight. The strength of the edge weights is shown on the x-axis. All possible edges are listed on the y-axis, ordered from highest (top) to lowest edge weight (bottom).

**Figure S2.** Difference test of edge weights for all edges significantly different from zero.

*Note.* Black boxes indicate significant differences between edge weights at *p* < .05.

**Figure S3**. Node centrality plot.

*Note.* The node centrality of the network that is presented in Figure 3 of the main manuscript, is calculated on the basis of connection strength as estimated in the *estimateNetwork* function. The centrality indices are shown as standardized z-scores. The node with the highest centrality index is (PR_G), The general value for participating in research. Nodes are ordered on connection strength. FH = Future Health; AL = Adapt Lifestyle to stay healthy; SM = Having a Say in Medical decisions; SD = Share medical Data to improve healthcare; PR = Participate in scientific Research to improve healthcare. K = Knowledge; B = Behavior; E = Emotion; S = Self-efficacy; P = Personal value; G = General value.

**Figure S4*.*** Difference test of strength centrality per node.

*Note.* Results of statistical tests for difference between strengths for all nodes based on bootstrap results (nBoots = 2000 in the function *bootnet*; Epskamp et al., 2017). Black boxes indicate significant differences between node strength at *p* < .05. The strength per node is displayed in the diagonal.

**Figure S5.** Results of the subsetting bootstrap for strength centrality.

*Note.* The average correlation between the strength of nodes sampled with cases dropped and the original network. On the x-axis the percentage of cases dropped and on the y-axis the correlation is represented. The line indicates the mean and area indicate the range from the 2.5th quantile to the 97.5th quantile.

**Performing network analysis.**

Free software (JASP), which includes options to perform network analyses (Van Doorn et al., 2021), can be downloaded from <https://jasp-stats.org/>. The network package can be added by clicking the blue plus in the upper right corner and selecting ‘Network’.

For a tutorial paper on estimating psychological networks and their accuracy, see Epskamp, Borsboom and Fried (2018). An instruction for performing network analysis in JASP is described in this blog post: <https://jasp-stats.org/2018/03/20/perform-network-analysis-jasp/> .

The interest data from the current study is available from OSF (<https://osf.io/c6bmx/?view_only=9e977b19a3754f7ab377ddb011bd1f9f> ), this will offer the possibility to practice network analysis with real data.

When performing the Network analysis in JASP, note that parameters used in this study are set as follows:

- Dependent Variables are in Scale,
- Estimator = mgm,
- Criterium = CV (Cross-validation),
- nFolds = 10,
- Variables in network = Continuous Variables

**Table S1.**

| Correlations (upper triangle) and partial correlations (lower triangle) between the topics and interest dimensions included in the interest in personalized medicine network. |
| --- |


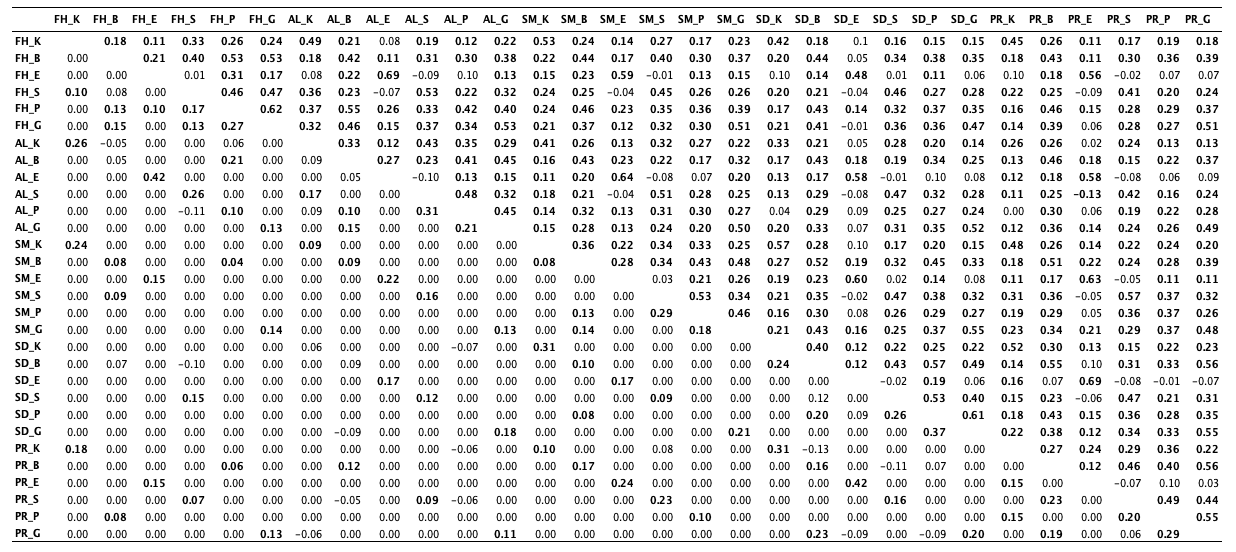


| *Note*. Values in bold denote significant pearson correlations (*p* < .05; upper triangle) or edges significantly different from zero-edges in the network based on a bootstrap difference test (lower triangle; see Figure S4 in the Supplementary Materials). |
| --- |
