# Supplementary figures and images for: Presenting wicked problems in a science museum: A methodology to study interest from a dynamic perspective
Source: Front Psychol. 2023 Feb 10;14:1113019. doi: 10.3389/fpsyg.2023.1113019 (PMC9951591; doi:10.3389/fpsyg.2023.1113019)

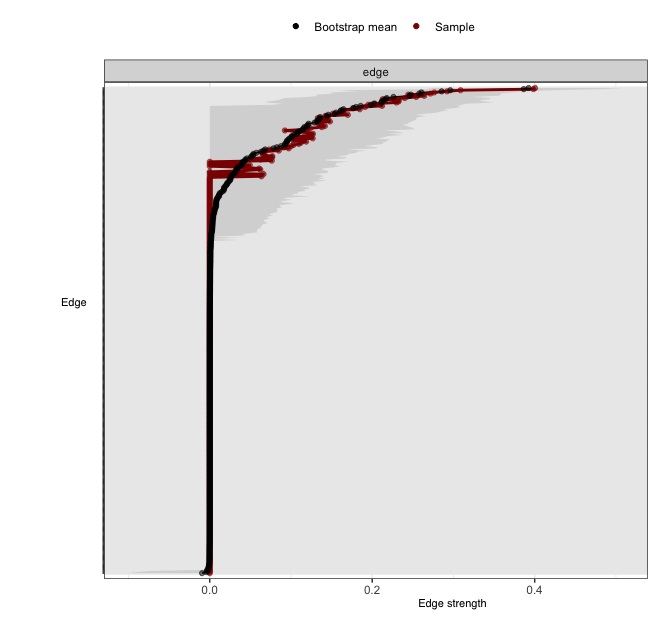

Supplement: Supplementary file 2 [file Image_1.JPEG]

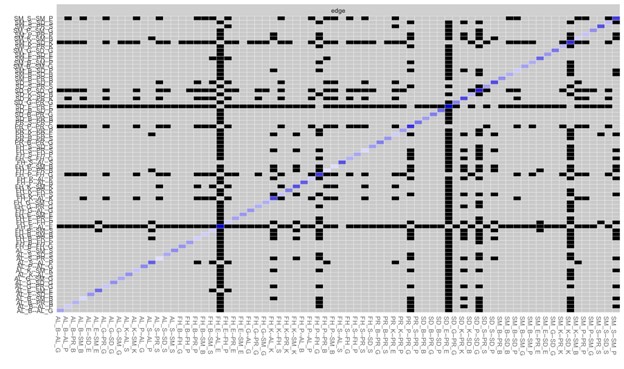

Supplement: Supplementary file 3 [file Image_2.JPEG]

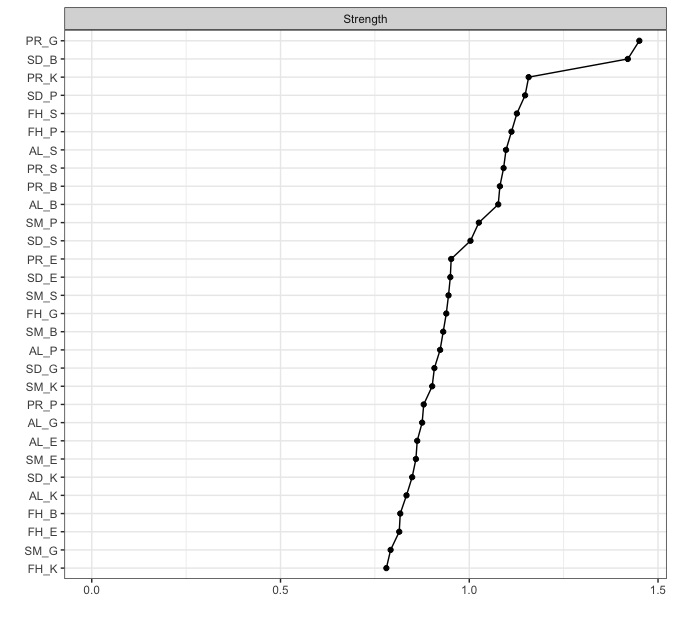

Supplement: Supplementary file 4 [file Image_3.JPEG]

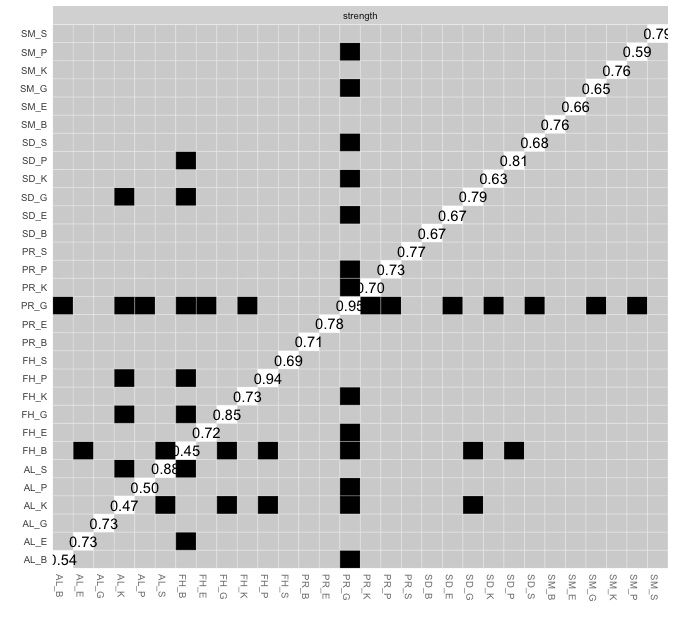

Supplement: Supplementary file 5 [file Image_4.JPEG]

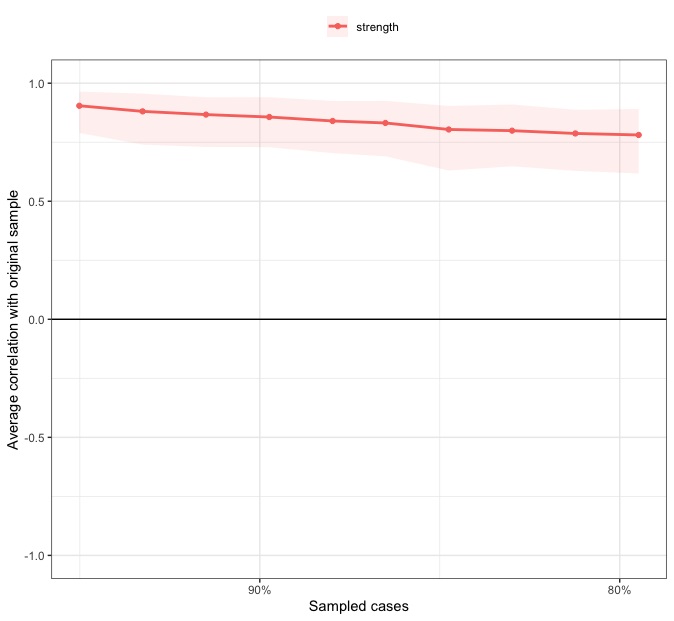

Supplement: Supplementary file 6 [file Image_5.JPEG]
